# Comparative analysis of soybean transcriptional profiles reveals defense mechanisms involved in resistance against *Diaporthe caulivora*

**DOI:** 10.1038/s41598-023-39695-1

**Published:** 2023-08-11

**Authors:** Eilyn Mena, Guillermo Reboledo, Silvina Stewart, Marcos Montesano, Inés Ponce de León

**Affiliations:** 1 Departamento de Biología Molecular, Instituto de Investigaciones Biológicas Clemente Estable, Montevideo, Uruguay; 2 Programa Nacional de Cultivos de Secano, Instituto Nacional de Investigación Agropecuaria (INIA), La Estanzuela, Colonia, Uruguay; 3 Laboratorio de Fisiología Vegetal, Centro de Investigaciones Nucleares, Facultad de Ciencias, Universidad de la República, Montevideo, Uruguay

**Keywords:** Molecular biology, Plant sciences

## Abstract

Soybean stem canker (SSC) caused by the fungal pathogen *Diaporthe caulivora* is an important disease affecting soybean production worldwide. However, limited information related to the molecular mechanisms underlying soybean resistance to *Diaporthe* species is available. In the present work, we analyzed the defense responses to *D. caulivora* in the soybean genotypes Williams and Génesis 5601. The results showed that compared to Williams, Génesis 5601 is more resistant to fungal infection evidenced by significantly smaller lesion length, reduced disease severity and pathogen biomass. Transcriptional profiling was performed in untreated plants and in *D. caulivora*-inoculated and control-treated tissues at 8 and 48 h post inoculation (hpi). In total, 2.322 and 1.855 genes were differentially expressed in Génesis 5601 and Williams, respectively. Interestingly, Génesis 5601 exhibited a significantly higher number of upregulated genes compared to Williams at 8 hpi, 1.028 versus 434 genes. Resistance to *D. caulivora* was associated with defense activation through transcriptional reprogramming mediating perception of the pathogen by receptors, biosynthesis of phenylpropanoids, hormone signaling, small heat shock proteins and pathogenesis related (PR) genes. These findings provide novel insights into soybean defense mechanisms leading to host resistance against *D. caulivora*, and generate a foundation for the development of resistant SSC varieties within soybean breeding programs.

## Introduction

Soybean (*Glycine max* L.) is a major global crop affected by biotic stress caused by microbial pathogens, nematodes and insects, as well as abiotic stress such as drought, nutrient deficiency, salt and cold^1^. Soybean stem canker (SSC) caused by fungal *Diaporthe* species is an important soybean disease worldwide. *D. aspalathi* (E. Jansen, Castl. & Crous)*, D. caulivora* (Athow & Caldwell) and *D. longicolla* (Hobbs) are the principal agents causing SSC in different countries^2–5^. Disease symptoms appear on the stem as 1–2 mm spots that expand as elongated brown lesions associated to withered brown leaves^3^. SSC control is based on integrating management practices such as crop rotation and fungicides application. However, the most effective way to control SSC is to develop and use resistant cultivars. Five *Rdm* loci confer resistance to *D. aspalathi*^3,6^, although these resistance loci are not effective against *D. caulivora*^3^. Recently, an *Rdc1* locus of *G. max* was identified as a resistance source for *D. caulivora*^7^, although the molecular identity of *Rdc1* is currently unknown.

Plants perceive pathogens and trigger cellular and molecular modifications associated with defense responses such as signaling, transcriptional activation, synthesis of defense molecules, and their transport to specific sites in the plant^8^. Recognition occurs at the plasma membrane by pattern-recognition receptors (PRRs), and in the cytoplasm by nucleotide-binding domain leucine-rich repeat containing receptors (NLRs)^9,10^. PRRs perceive conserved microbe- or damage-associated molecular patterns (MAMPs or DAMPs) at the surface of the plant cells to activate pattern-triggered immunity (PTI). PRRs include receptor-like kinases (RLKs) or receptor-like proteins (RLP) with different extracellular domains. NLRs perceive pathogen effectors delivered inside the plant cell leading to effector-triggered immunity (ETI). Both PTI and ETI activate overlapping events such as mitogen-activated protein kinases (MAPKs) cascades, Ca^2+^ flux, hormonal signaling, reactive oxygen species (ROS) burst, callose deposition, and transcriptional reprogramming^11,12^.

During soybean-*D. caulivora* interaction, plant cells activate the expression of genes encoding pathogenesis-related proteins (PR-1, PR-2, PR-3, PR-4, PR-10), and enzymes involved in phenylpropanoid and oxylipin synthesis such as phenylalanine-ammonia lyase (PAL), chalcone synthase (CHS), and lipoxygenase (LOX)^2^. Most of these defense genes were also induced in soybean tissues infected with *D. aspalathi*^13^. The phenylpropanoid and oxylipin pathways participate in plant defenses against pathogens by producing important compounds with antimicrobial activities, and contribute to reinforcement of the plant cell walls and defense signaling^14,15^. Recently, we sequenced the genome of *D. caulivora* (isolate D57) and performed transcriptional profiling during stem colonization to reveal the molecular basis of fungal pathogenesis^16^. In this analysis, we identified a high number of fungal upregulated genes that encode enzymes involved in plant cell wall degradation and modification such as polygalacturonases, endoglucanases, exoglucanases, pectate lyases, pectin lyases, and glycoside hydrolases, among others^16^. *D. caulivora* infection strategy also relies on detoxification of plant compounds, transporter activities, and toxin production that could kill plant cells, enabling nutrient uptake and mycelium growth^16^. Moreover, *D. caulivora* genes encoding secreted effector candidates are induced during soybean infection, suggesting that plant defense evasion contributes to the plant colonization process^16^. However, the molecular mechanisms employed by plant cells to recognize *D. caulivora* and activate an effective defense response leading to resistance are still unknown.

Transcriptomic studies have allowed to understand complex gene regulatory networks operating in soybean plants infected with different pathogens, including *Phytophthora sojae* (Kaussmann & Gerdemann)^17^, *Phakopsora pachyrhizi* (Sydow & P. Sydow)^18,19^, *Fusarium oxysporum* (Schltdl.)^20^, and soybean mosaic virus (SMV)^21^. To unravel the mechanisms involved in defense activation and resistance against *D. caulivora*, we performed RNAseq profiling in two contrasting soybean genotypes, the susceptible cultivar Williams and the resistant cultivar Génesis 5601. The results revealed a complex and differential gene expression network during the activation of plant defense responses between cultivars upon pathogen infection.

## Results

### *D. caulivora* infection of soybean genotypes

**Figure 1:**
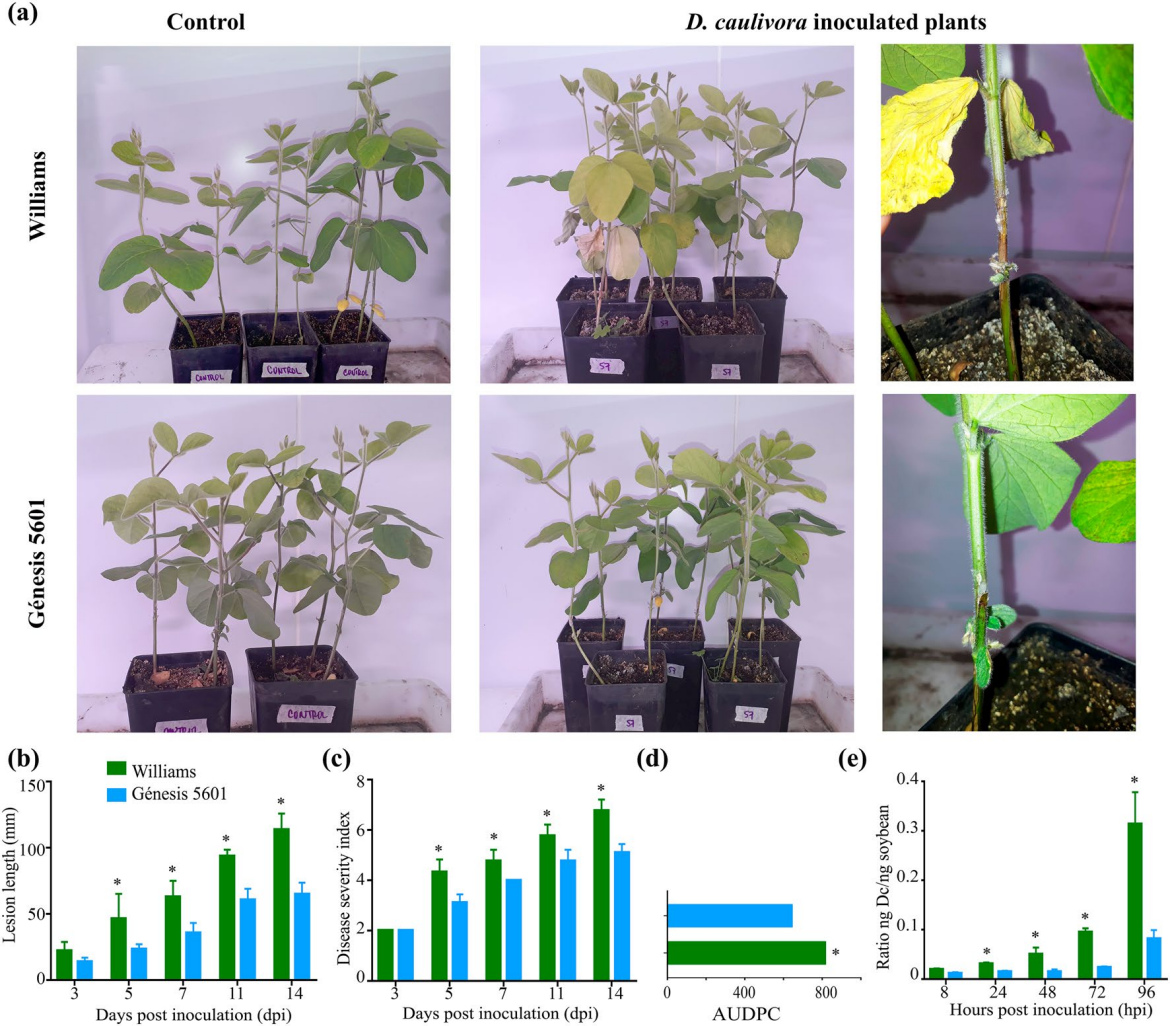
Soybean stem canker disease progress after *D. caulivora* inoculation. (**a**) Symptoms in susceptible and resistant soybean plants following *D. caulivora* inoculation at 7 days post-inoculation (dpi), (**b**) Lesion length in susceptible and resistant soybeans at 3, 5, 7, 11 and 14 dpi, (**c**) Disease severity index in susceptible and resistant soybeans at 3, 5, 7, 11 and 14 dpi, (**d**) AUDPC in susceptible and resistant soybeans at 3, 5, 7, 11 and 14 dpi. *D. caulivora* biomass in susceptible and resistant soybeans at 8, 24, 48, 72 and 96 hpi. Asterisk indicates a significant difference between the soybean genotypes at p-value < 0.05 (one-way ANOVA).

Williams and Génesis 5601 cultivars were inoculated with *D. caulivora* and stem canker progress was monitored during 14 days post inoculation (dpi). The first symptoms of SSC were observed at 3 dpi, and lesions were more evident at 5 dpi showing typical stem browning (Supplementary Fig. S1). Lesions expanded in both directions of the stem and disease symptoms were clearly visible in Williams, while Génesis 5601 exhibited smaller stem lesions (Fig. 1a, Supplementary Fig. S1). Moreover, withered leaves above de canker lesion were only observed in Williams (Fig. 1a). From 5 to 14 dpi, lesion length was significantly higher in Williams compared to Génesis 5601, varying between 36 and 50% (Fig. 1a, b). Disease severity index and area under the disease progress curve (AUDPC) were evaluated in soybean stems until 14 dpi. According to a disease severity scale^2^, *D. caulivora* infection resulted in more symptom development in Williams compared to Génesis 5601 throughout time (Fig. 1c, d).

We previously showed that *D. caulivora* biomass starts to increase at 8 hpi in Williams stems, and at 96 hpi fungal DNA became predominant in the host tissues^2^. Here, we measured by quantitative PCR (qPCR) *D. caulivora* biomass in stems of both genotypes at 8, 24, 48, 72 and 96 h post inoculation (hpi). The results show that pathogen biomass was significantly higher in Williams compared to Génesis 5601 at 24 to 96 hpi (2–4 fold) (Fig. 1e). Taken together, these results indicate that Génesis 5601 is more resistant to *D. caulivora* infection than Williams.

### Transcriptome profiles of contrasting soybean genotypes infected with *D. caulivora*

To identify molecular components involved in soybean defense responses against *D. caulivora*, we first compared the transcriptomes of Williams and Génesis 5601 under normal conditions in untreated plants. A second comparison included Williams and Génesis 5601 tissues inoculated with *D. caulivora* versus control treatment. Two time points, 8 and 48 hpi were selected based on previous expression patterns of PRs, PAL, CHS and LOX in *D. caulivora*-infected soybean plants^2^. In total, 819 million reads were generated after removing adapter sequences and low-quality reads. Reads mapped uniquely to the *G. max* nuclear reference genome (approximately 737 million reads) were considered for further analyses (Supplementary Table S1). The principal component analysis of the different treatments showed a clear separation, and variability among biological replicates was very low as indicated (Supplementary Figure S2).

**Figure 2:**
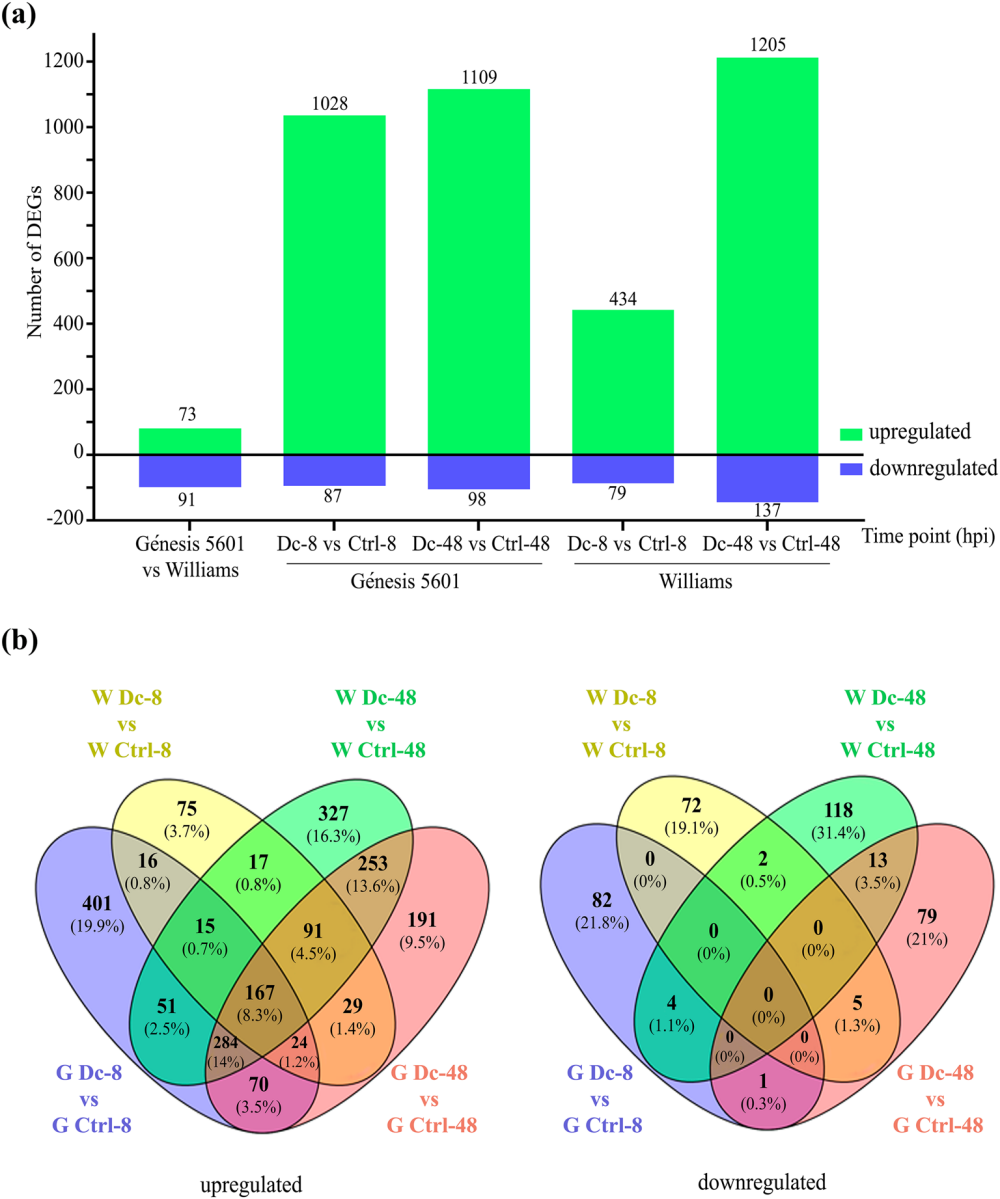
Differentially expressed genes (DEGs) identification in susceptible (Williams) and resistant (Génesis 5601) soybean plants without treatment and after *D. caulivora* inoculation. (**a**) Number of upregulated and downregulated DEGs for each treatment in both genotypes. Log2 FC ≥ 2.0 or ≤ 2.0 and false discovery rate (FDR) ≤ 0.05 were considered for DEGs identification, (**b**) Venn diagram showing the number of upregulated and downregulated soybean genes at 8 and 48 h post inoculation (hpi) with *D. caulivora* in susceptible and resistant soybean plants. Overlap of expressed soybean genes are indicated by bold numbers. See Supplementary Table S2 for complete information.

Soybean expression profile analysis identified 2.384 differentially expressed genes (DEGs) between soybean genotypes (Supplementary Table S2). A significant transcriptional shift towards upregulation was observed at 8 and 48 hpi after *D. caulivora* inoculation compared to control treatment in both cultivars (from now on named as Génesis-8 and -48 and Williams-8 and -48) (Fig. 2a). Untreated Génesis 5601 plants exhibited 164 DEGs (73 up- and 91 downregulated) compared to untreated Williams plants. During infection, Williams-48 has more DEGs than Williams-8, 1.342 and 513, respectively (Fig. 2a, Supplementary Table S2). However, the number of DEGs in Génesis-8 and Génesis-48 were similar, 1.115 and 1.207, respectively, indicating that Génesis-8 has significantly more DEGs than Williams-8. Among DEGs, 167 were upregulated in both genotypes at both time points, while some genes were uniquely expressed in one condition (Fig. 2b). Interestingly, 335 DEGs (32,6%) that were upregulated in Génesis-8 and not in Williams-8, were upregulated in Williams-48, indicating that these genes were later expressed in the susceptible genotype. In total, 930 DEGs were commonly upregulated in both genotypes, representing 46% of the upregulated DEGs (2.011 genes). In contrast, 22 DEGs were downregulated in both genotypes, representing 5.9% of the downregulated DEGs (376 genes). Furthermore, 75 and 327 upregulated DEGs were only found in Williams-8 and Williams-48, and 401 and 191 upregulated DEGs were only present in Génesis-8 and Génesis-48, respectively (Fig. 2b). Thus, the presence of a significantly higher number of upregulated DEGs in Génesis-8 compared to Williams-8 could be related to the molecular mechanisms involved in resistance against *D. caulivora*.

### Functional enrichment of differentially expressed genes

**Figure 3:**
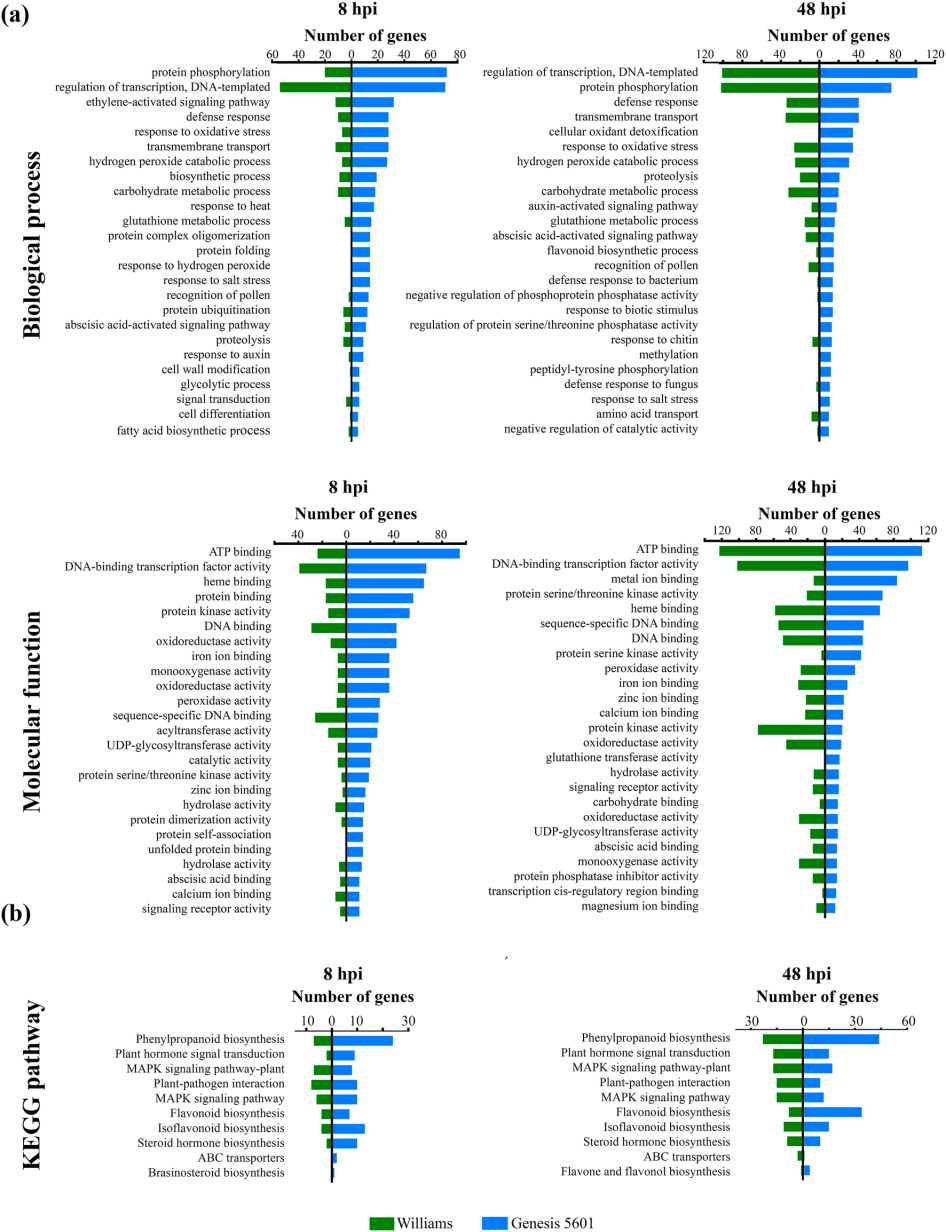
Enriched gene ontology (GO) and KEGG pathways analysis of upregulated genes in soybean plants inoculated with *D. caulivora*. (**a**) Top 25 enrichment GO biological process and molecular function terms (p < 0.05) of upregulated genes in Williams (green bars) and Génesis 5601 (blue bars) at 8 and 48 h post inoculation (hpi) with *D. caulivora*. See Supplementary Table S2 for complete information. (**b**) Upregulated genes KEGG pathway analysis in Williams (green bars) and Génesis 5601 (blue bars) at 8 and 48 h post inoculation (hpi) with *D. caulivora*. See Supplementary Table S3 for complete information.

In order to identify biological processes (BP) and molecular functions (MF) mostly affected by *D. caulivora* infection, we performed Gene Ontology (GO) term enrichment analysis of the upregulated DEGs. Enriched GO were not found for untreated Williams or Génesis 5601 tissues. Most of the top 25 significantly enriched GO terms in *D. caulivora*-inoculated versus control soybean plants were similar at 8 and 48 hpi (Fig. 3a, Supplementary Table S2). Génesis 5601 showed a significantly higher number of genes per category respect to Williams, principally at 8 hpi. The most represented BP in both genotypes at 8 and 48 hpi were protein phosphorylation, regulation of transcription, defense response, and transmembrane transport, among others. Other top GO terms enrichment in BP at 8 hpi included ethylene-activated signaling pathway, response to oxidative stress and hydrogen peroxide, response to heat and salt stress, ABA activated signaling pathway, response to auxin and cell wall modification. Most of these enriched BP related to defense were also present at 48 hpi, and additional upregulated defense-related GO terms at 48 hpi included cellular oxidant detoxification, flavonoid biosynthetic process, and response to biotic stimulus, bacterium, chitin and fungus. In general, biotic related process, abiotic related process, hormones and secondary metabolites represented 60% of the BP terms identified in the upregulated genes. In both genotypes, MF terms at 8 and 48 were represented by ATP binding, DNA-binding transcription factor activity, heme- , protein- , DNA- and iron ion-binding, sequence-specific DNA binding, protein kinase- , oxidoreductase- , monooxygenase- , peroxidase- , glycosyltransferase- and protein serine threonine kinase-activity.

To study the host pathways altered during *D. caulivora* infection, we performed a KEGG enrichment analysis (Fig. 3b, Supplementary Table S3). The most enriched KEGG pathways for upregulated DEGs were related to phenylpropanoid, flavonoid and isoflavonoid biosynthesis, plant hormone signal transduction, mitogen-activated protein kinase (MAPK) signaling, plant-pathogen interaction, and steroid hormone biosynthesis. Both genotypes shared most of the KEGG pathways, although there were more genes within pathways associated to Génesis 5601 compared to Williams.

### Differential expression of genes involved in plant defense during *D. caulivora* infection

**Figure 4:**
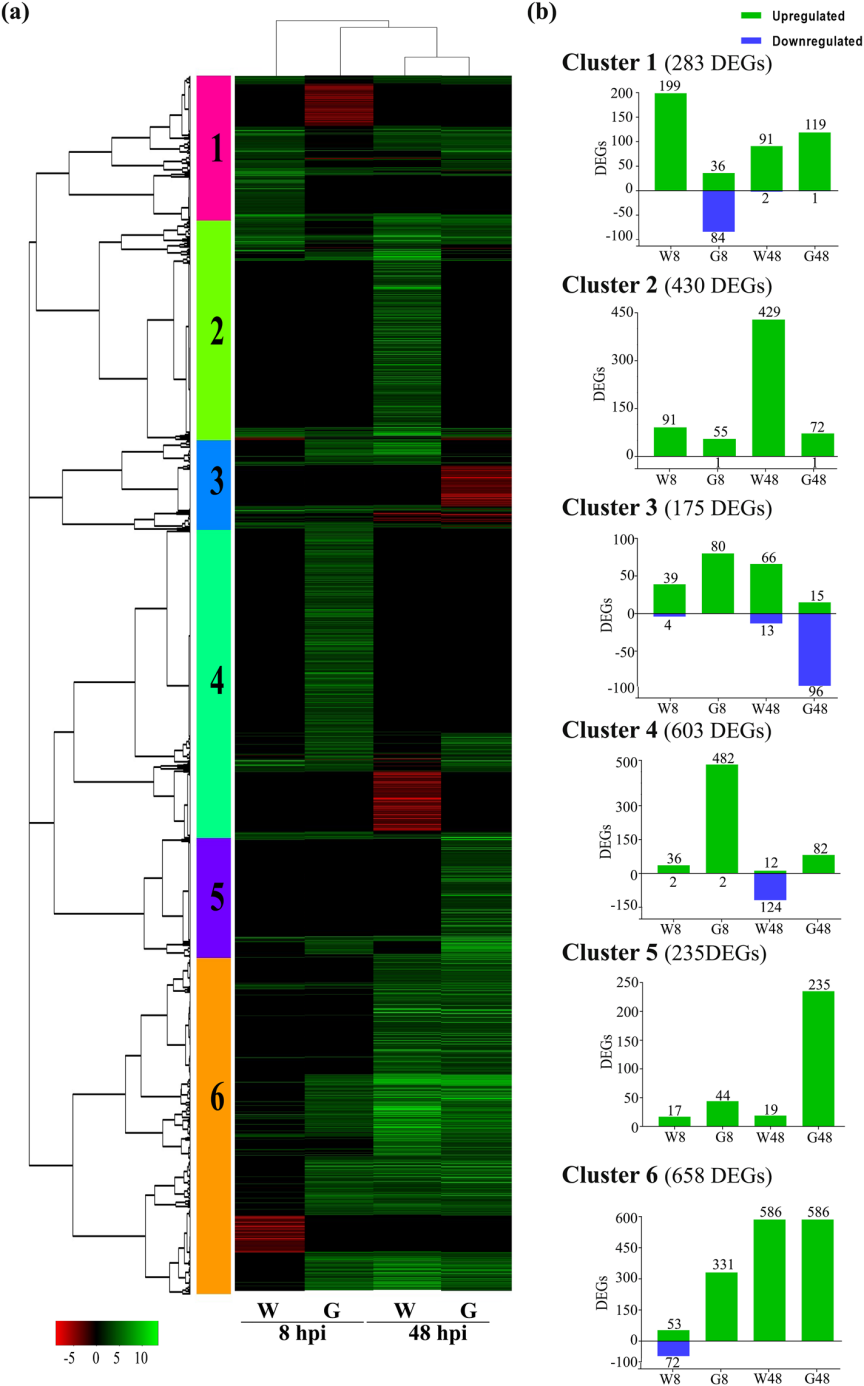
Differentially expressed genes (DEGs) in soybean plants inoculated with *D. caulivora*. **(a)** Heat map of hierarchical clustering of all DEGs. Green represents upregulated DEGs and red downregulated DEGs. (**b**) Numbers of total, upregulated and downregulated DEGs for each cluster. See Supplementary Table S4 for complete information.

Hierarchical clustering was performed to group similar expression patterns across genotypes and treatments. This analysis grouped Williams-48 with Génesis-48, and Génesis-8 was more related to this group than to Williams-8, which is consistent with a significantly higher number of upregulated DEGs in Génesis-8 (Fig. 4, Supplementary Table S4). The analysis of total DEGs identified six clusters with different gene numbers and expression patterns (Fig. 4a, b). Clusters 4, 5, and 6 were strongly associated with increased expression of genes in Génesis-8 and Génesis-48 compared to the same time points in Williams. Génesis-8 contained 482 and 331 upregulated genes in cluster 4 and 6 respectively, while Génesis-48 comprises 235 upregulated DEGs in cluster 5. The corresponding numbers of upregulated DEGs in the Williams genotype were 36, 53 and 19 DEGS (Fig. 4b). To explore the BP associated with Génesis 5601 resistance, GO terms enrichment analysis was performed for DEGs present in the different clusters (Supplementary Table S5). Common GO terms identified in most clusters included regulation of DNA-templated transcription, protein phosphorylation, defense response, transmembrane transport, flavonoid or isoflavonoid biosynthetic process, among others. Other major GO terms associated with Génesis-8 in cluster 4, included response to hydrogen peroxide, response to oxidative stress, hydrogen peroxide catabolic process, glutathione metabolic process, response to salt stress and response to heat stress. Response to oxidative stress and cellular oxidant detoxification were also GO terms enriched in cluster 5 and 6, and the latter comprises other GO terms related to hormones. By performing a deeper inspection of genes within GO terms among genotypes, we found that the term response to heat stress in cluster 4 included DEGs that encode small heat shock proteins (sHSPs) that were only upregulated in Génesis 5601, while they did not show differential expression in Williams. In total, 17 sHSPs and one sHSP were upregulated in Génesis-8 and Génesis-48, respectively. Taken together, these results suggest that regulation of transcription, signaling, phenylpropanoid and flavonoid pathways, ROS detoxification and sHSPs play important functions in plant resistance against *D. caulivora*.

### Induced expression of genes involved in pathogen perception, signaling and transcription during soybean infection by *D. caulivora*

**Figure 5:**
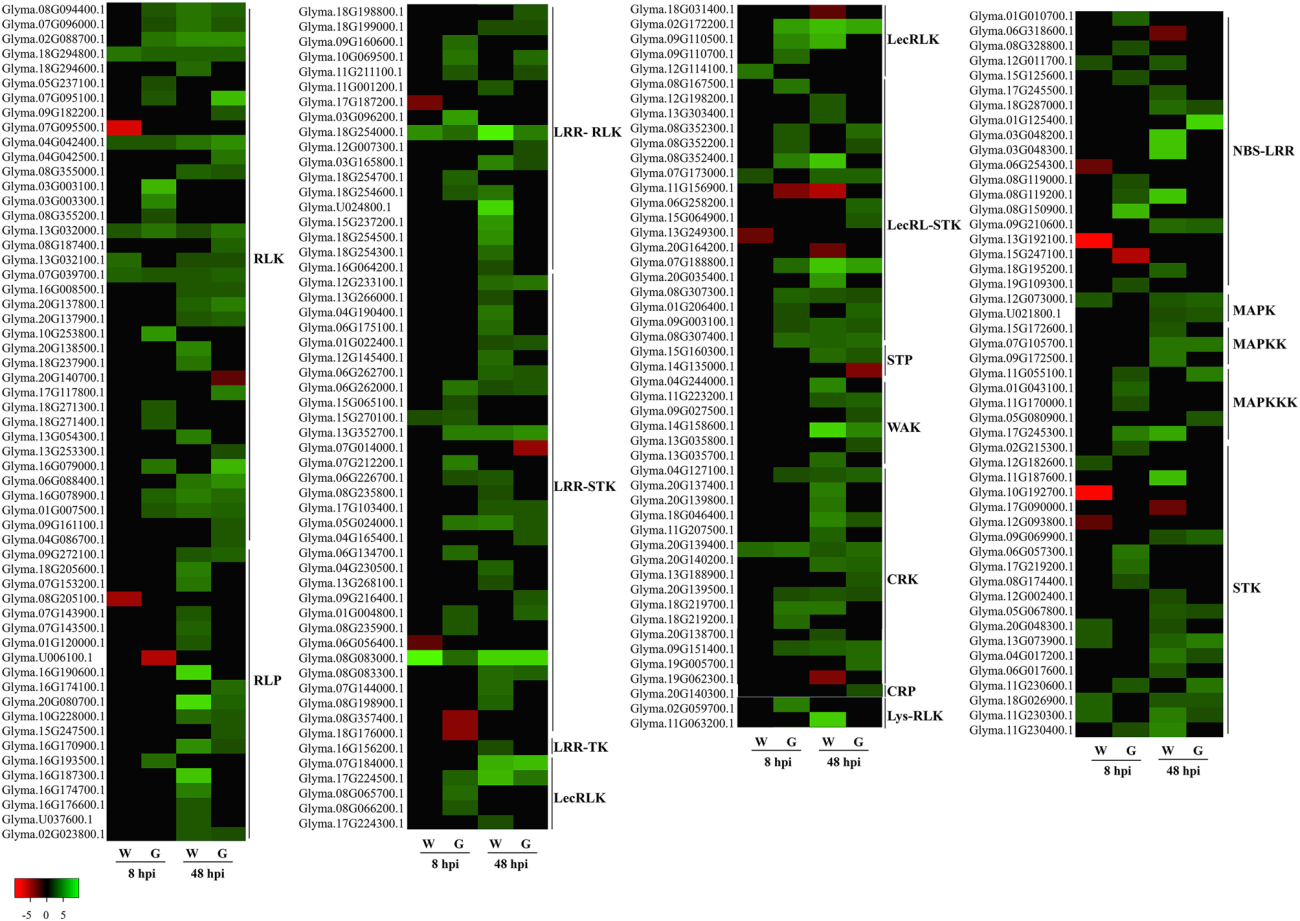
Heat map of differentially expressed genes (DEGs) encoding for proteins with roles in perception and signaling. Individual genes are listed and colors represent the log2 fold change value based on the comparison of the transcript levels between *D. caulivora*-inoculated and control treatment for both genotypes (Williams and Génesis 5601). Green represents upregulated DEGs and red downregulated DEGs. *RLK* receptor-like protein kinase, *RLP* receptor-like protein, *LRR-RLK* leucine-rich repeat receptor-like protein kinase, *LecRLK* lectin domain containing receptor kinase, *WAK* wall-associated receptor kinase, *CRK* cysteine-rich receptor-like protein kinase, *CRP* cysteine-rich protein, *LysM-RLK* LysM domain receptor-like kinase, *NLR* nucleotide-binding site leucine-rich repeat, *MAPK* mitogen-activated protein kinase, *MAPKK* mitogen-activated protein kinase kinase, *MAPKKK* mitogen-activated protein kinase kinase kinase, and *STK* serine/threonine-protein kinase. See Supplementary Table S6 for complete information.

Pathogen recognition, signaling and transcriptional reprogramming are important steps in the activation of plant defense mechanism against pathogens. We identified 159 DEGs encoding PRRs, most of which were upregulated during *D. caulivora* infection, including leucine-rich repeat receptor-like protein kinase (LRR- RLK), RLKs, RLPs and lectin domain containing receptor kinase (LecRLKs), among others (Fig. 5, Supplementary Table S6). While only 11 receptor genes were upregulated in Williams-8, this number increased to 59 in Génesis-8, and included LRR-RLKs, RLPs, cysteine-rich receptor-like protein kinase (CRKs) and LecRLKs. At 48 hpi, 76 and 94 receptor genes were upregulated in Génesis-48 and Williams-48, respectively. Furthermore, 15 upregulated DEGs encoded NLR; seven in Génesis-8, one in Williams-8, three in Génesis-48 and eight in Williams-48. In untreated plants, a higher number of PRRs and NLR genes showed increased expression levels in Génesis 5601 compared to Williams.

**Figure 6:**
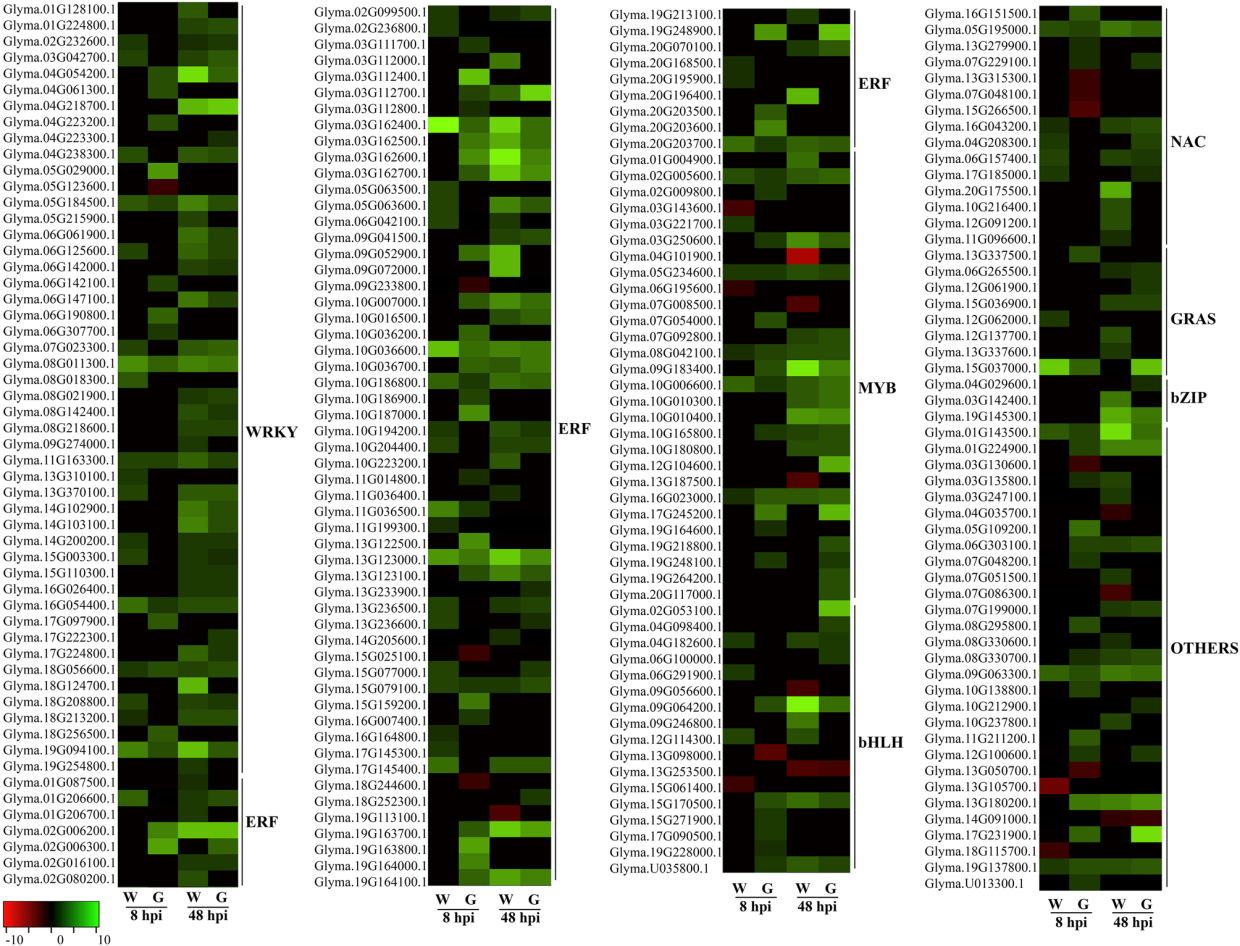
Heatmap of differentially expressed genes (DEGs) encoding for transcription factors. Individual genes are listed and colors represent the log2 fold change value based on the comparison of the transcript levels between *D. caulivora*-inoculated and control treatment for both genotypes (Williams and Génesis 5601). Green represents upregulated DEGs and red downregulated DEGs. *ERF* ethylene responsive transcription factor, *MYB* myeloblastosis-related transcription factor, *bHLH* basic helix–loop–helix transcription factor, *NAC* no apical meristem, ATAF1/2, and cup shaped cotyledon, *GRAS* gibberellin-insensitive, repressor of GA1-3 and Scarecrow, basic region/leucine zipper motif (bZIP). See Supplementary Table S7 for complete information.

Thirty DEGs encoded members of MAPK cascades, including MAPK, MAPKK and MAPKKK and serine/threonine-protein kinase (STKs) (Fig. 5, Supplementary Table S6). Four MAPKKK were only induced in Génesis-8 and different STKs were upregulated in Génesis-8 and Williams-8. Moreover, 219 DEGs encoded TFs related to plant defenses to biotic stress, including 48 WRKYs, 71 Apetala2/ethylene responsive factor (AP2/ERFs), 28 myeloblastosis-related (MYB), 17 basic helix–loop–helix (bHLH), 15 no apical meristem, ATAF1/2, and cup-shaped cotyledon (NAC), and eight Gibberellin-insensitive, repressor of GA1–3, and Scarecrow (GRAS) (Fig. 6, Supplementary Table S7). Most TFs were upregulated during *D. caulivora* infection and the number of upregulated TFs increased significantly at 48 hpi respect to 8 hpi in both genotypes. Génesis-8 exhibited more upregulated *ERFs*, *MYBs* and *bHLHs* compared to Williams-8.

### Activation of pathogenesis-related genes and the phenylpropanoid pathway during *D. caulivora* infection

Pathogenesis-related proteins play important functions in plant immune responses^22^. During *D. caulivora* infection, soybean induced the expression of 139 PR genes encoding PR-1, β-1,3-glucanases (PR-2), chitinases (PR-3, PR-4, PR-8), thaumatins (PR-5), proteinase inhibitors (PR-6), endoproteinasse (PR-7), peroxidases (PR-9), PR-10 (ribonuclease-like protein), defensins (PR-12), lipid-transfer proteins (PR-14) and germin-like proteins (PR-16) (Supplementary Fig. S3, Supplementary Table S8). A higher number of genes encoding PRs were upregulated in Génesis-8 compared to Williams-8, while at 48 hpi the number of upregulated genes were similar among genotypes. Four PR-2 were only induced in Génesis-8 and not in Williams-8, while six PR-14 were induced in Génesis-8 and only one in Williams-8. Similarly, 27 PR-9 and 10 PR-10 were upregulated in Génesis-8, while this number decreased to seven and four in Williams-8.

**Figure 7:**
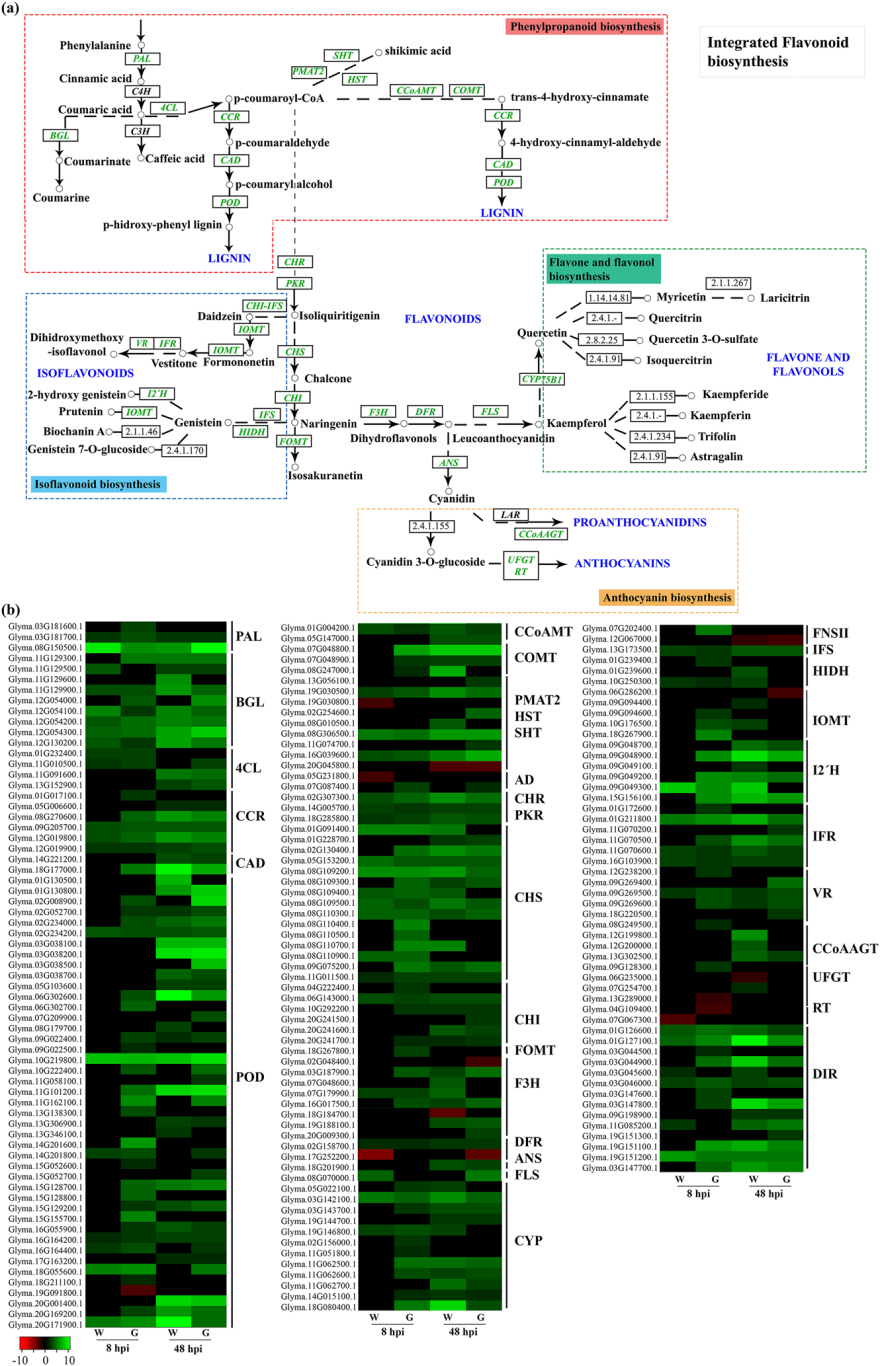
Activation of the phenylpropanoid and flavonoids pathways in response to *D. caulivora*. (**a**) Integrated and simplified scheme of phenylpropanoid, flavonoid, isoflavonoid, anthocyanin, flavone and flavonol biosynthesis KEGG pathways. Upregulated genes encoding enzymes of this pathway are highlighted in green. (**b**) Heatmap of DEGs encoding genes of the phenylpropanoid and flavonoids biosynthetic pathway. Individual genes are listed and colors represent the log2 fold change value based on the comparison of the transcript levels between *D. caulivora*-inoculated and control treatment for both genotypes (Williams and Génesis 5601). Green represents upregulated DEGs and red downregulated DEGs. *PAL* phenylalanine ammonia-lyase, *BGL* beta-glucosidase, *4CL* 4-coumarate–CoA ligase, *CCR* cinnamoyl-CoA reductase, *CAD* cinnamyl alcohol dehydrogenase, *POD* peroxidase, *CCoAMT* caffeoyl-CoA *O*-methyltransferase, *COMT* caffeic acid 3-*O*-methyltransferase, *PMAT2* phenolic glucoside malonyltransferase 1-like, *HST* spermidine hydroxycinnamoyl transferase, *SHT* shikimate *O*-hydroxycinnamoyl transferase, *AD* aldehyde dehydrogenase, *CHR* chalcone reductase, *CHS* chalcone synthase, *CHI* chalcone isomerase, *FOMT* isoflavone 7-*O*-methyltransferase, *F3H* flavanone 3-hydroxylase, *DFR* dihydroflavonol-4-reductase, *ANS* 2-oxoglutarate-dependent dioxygenase, *FLS* DMR6-like oxygenase 2, *CYP* cytochrome P450, *FNSII* flavone synthase II, *IFS* isoflavone synthase 2, *HIDH* 2-hydroxyisoflavanone dehydratase, *IOMT* isoflavone 7-*O*-methyltransferase, *I2'H* isoflavone 2'-hydroxylase, *IFR* isoflavone reductase, *VR* vestitone reductase, *CCoAAGT* coumaroyl-CoA: anthocyanidin 3-*O*-glucoside-6''-*O*-coumaroyltransferase, *UFGT* UDP-glycosyltransferase, *RT* UDP-rhamnose: rhamnosyltransferase, *DIR* Dirigent protein. See Supplementary Table S9 for complete information.

The phenylpropanoid pathway produces multiple compounds involved in defense mechanisms against biotic stress^14^. In total, 169 DEGs related to this pathway were identified during *D. caulivora* infection. A high number of genes encoding enzymes involved in flavonoids, isoflavonoids, flavonone, flavonols, flavones and anthocyanins biosynthesis were upregulated in both genotypes at 48 hpi (Fig. 7, Supplementary Table S9). The most remarkable differences between genotypes were observed in Génesis-8, which has significantly more upregulated DEGs than Williams-8 (117 versus 50). For example, while 10 cytochrome P450 (CYP) and 14 CHS encoding genes were induced in Génesis-8, this number decreased to two *CYPs* and eight *CHSs* in Williams-8. Similarly, Génesis-8 showed increased expression of genes encoding for three isoflavone 7-*O*-methyltransferases (IOMT) and four isoflavone 2'-hydroxylases (I2¨H), while Williams-8 showed only increased expression of one *I2¨H* and differential expression of IOMT genes could not be observed. Other genes encoding dirigent proteins, involved in the synthesis of lignans and lignin, were also upregulated during *D. caulivora* infection in both genotypes.

### Plant hormones involved in soybean defense against *D. caulivora*

**Figure 8:**
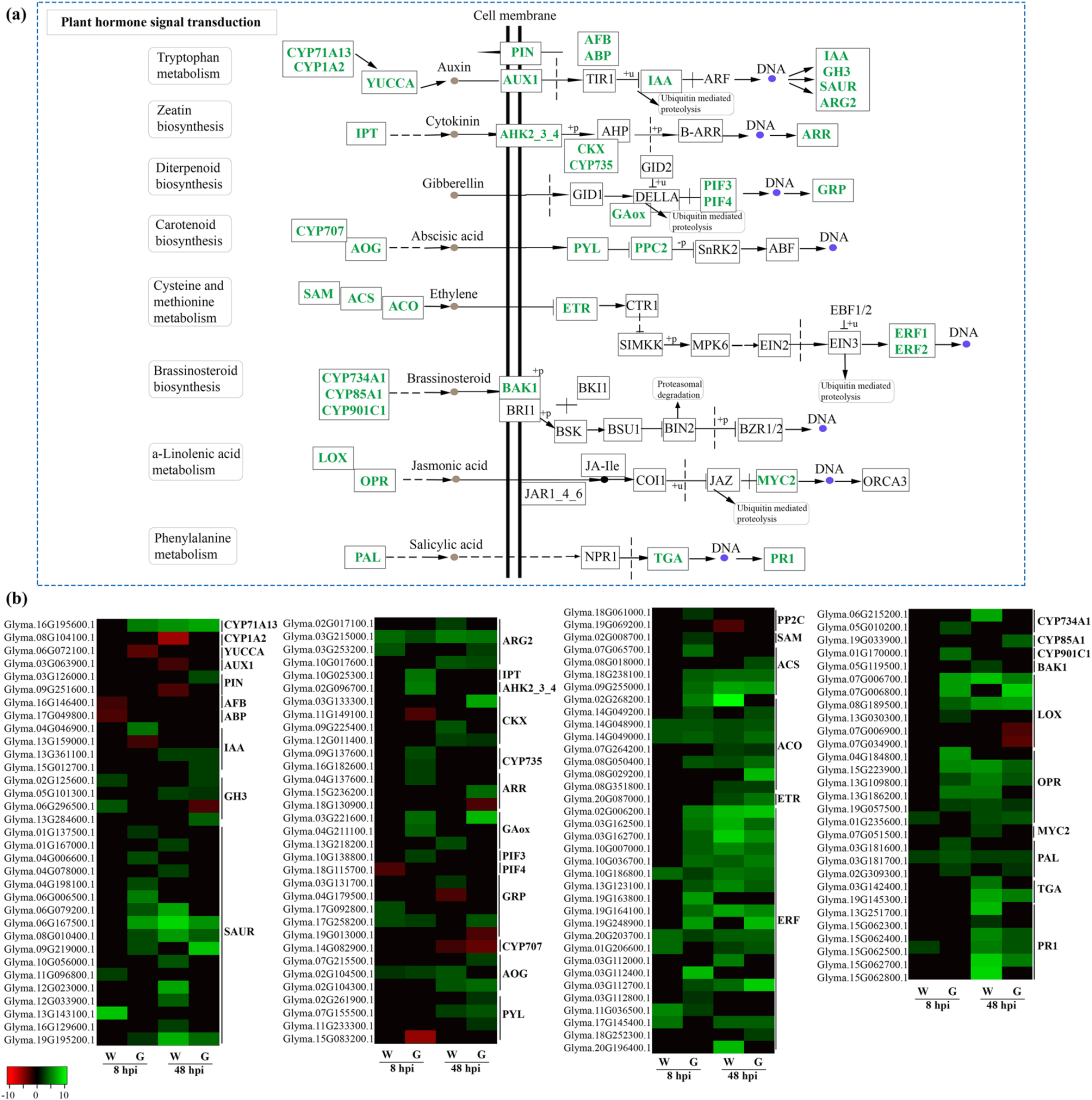
Activation of plant hormone signal transduction pathways after *D. caulivora* inoculation. (**a**) Simplified scheme of KEGG hormones signaling. Upregulated genes are represented in green. (**b**) Heatmap of DEGs encoding enzymes involved in defense hormone signaling. Individual genes are listed and colors represented the log2 fold change value based on the comparison of the transcript levels between *D. caulivora* inoculated and control treatment for both genotypes (Williams and Génesis 5601). Green represents upregulated DEGs and red downregulated DEGs. *CYP71A13, CYP1A2* cytochrome P450, *YUCCA* indole-3-pyruvate monooxygenase YUCCA, *AUX1* auxin influx carrier, *PIN* auxin efflux carrier, *AFB* auxin signaling F-box, *ABP* auxin-binding protein, *IAA* auxin responsive protein, *GH3* auxin responsive GH3 gene family, *SAUR* SAUR family protein, *ARG2* indole-3-acetic acid-induced protein ARG2, *IPT* adenylate isopentenyltransferase, *AHK2_3_4* Arabidopsis histidine kinase 2/3/4, cytokinin receptors, *CKX* cytokinin dehydrogenase, *CYP735* cytokinin hydroxylase, *ARR* two-component response regulator ARR family, *GAox* gibberellin oxidase, *PIF* phytochrome-interactin factor, *GRP* gibberellin-regulated protein, *CYP707* abscisic acid 8'-hydroxylase, *AOG* abscisate beta-glucosyltransferase, *PYL* abscisic acid receptor PYR/PYL family, *PP2C* protein phosphatase 2C, *SAM S*-adenosylmethionine synthase, *ACS* 1-aminocyclopropane-1-carboxylate synthase, *ACO* 1-aminocyclopropane-1-carboxylate oxidase, *ETR* ethylene receptor, *ERF* ethylene-responsive transcription factor, *CYP734A1, CYP85A1, CYP901C1* cytochrome P450, *BAK1* brassinosteroid insensitive 1-associated receptor 1, *LOX* linoleate lipoxygenase, *OPR* 12-oxophytodienoate reductase, *MYC2* transcription factor MYC2, *PAL* phenylalanine ammonia-lyase, *TGA* transcription factor TGA, *PR-1* pathogenesis-related protein 1. See Supplementary Table S10 for complete information.

GO enrichment analysis and overrepresented DEGs showed that salicylic acid (SA), auxin, ET, jasmonic acid (JA), abscisic acid (ABA), cytokinins (CK) and brassinosteroids (BR) probably participate in defense responses against *D. caulivora*. In total, 131 DEGs were involved in phytohormone pathways. Three PAL-encoding genes with possible roles in SA synthesis were upregulated during *D. caulivora* infection, and two of these *PAL* genes were only upregulated in Génesis 5601. However, isochorismate synthase genes (*ICSs*) were not induced after *D. caulivora* inoculation (Fig. 8, Supplementary Table S10). Auxin-related DEGs included 37 genes involved in biosynthesis, signaling and response to indole-3-acetic acid (IAA), and most of them were upregulated. A higher number of small auxin-up RNA (SAUR) genes were induced in Génesis-8 and in Williams-48 (nine and 10), compared to two SAUR genes in Williams-8 and four in Génesis-48 (Fig. 8, Supplementary Table S10). Furthermore, significantly more upregulated DEGs involved in CK synthesis and signaling were observed in Génesis-8 compared to Williams-8. We found three genes encoding abscisate beta-glucosyltransferases, and three PYL4 ABA receptors that were upregulated with *D. caulivora*. Moreover, one ET receptor (ETR) gene was upregulated in both genotypes at 48 hpi, and 34 DEGs were related to ET synthesis and signaling; 24 were upregulated in Génesis-8 and only seven in Williams-8. Most of these DEGs encode 1-aminocyclopropane-1-carboxylate synthases (ACC), 1-aminocyclopropane-1-carboxylate oxidases (ACO) and ERF TFs. Nine upregulated DEGs encoded proteins involved in JA biosynthesis and signaling in Génesis-8 such as LOX, 12-oxophytodienoate reductase (OPR) and MYC2 TF, while in Williams-8 only one *OPR* was upregulated. At 48 hpi, the number of DEGs related to ET and JA pathways were similar among soybean genotypes. Taken together, these results suggest that several hormones, in particular IAA, ET, CK and JA are involved in resistance mechanisms against *D. caulivora*.

### Expression analysis of selected candidate genes by RT-qPCR

Twenty-four candidate genes were selected for qRT-PCR validation of the differential response observed in the transcriptomic analysis (Supplementary Fig. S4). Genes encoding proteins with important functions in plant defense against pathogens were included; five RLKs, two NLR, five TFs, one HSP70, three sHSP, four PRs, a CHS, one dirigent, one Bcl-2-associated athanogene (BAG) cochaperone, and a beta-glucosidase. Relative transcript accumulation observed by qPCR showed a strong correlation with transcriptomic expression profiles (R2 = 0.9502), validating the RNA-seq data (Supplementary Table S11).

## Discussion

Host responses to biotic stress rely on the timely recognition of the pathogen and the efficient activation of a defense response that involves transcriptional reprogramming. The first stages of the interaction are decisive for the outcome of the disease. Hence, we focused on two early stages of *D. caulivora* infection, 8 and 48 hpi, according to stem colonization process and induction of PR gene expression^2^. Comparative transcriptional profiles between Génesis 5601 and Williams detected more than 2.000 DEGs. From these, 46% were commonly upregulated in both genotypes during *D. caulivora* infection, indicating overlapping responses. Interestingly, Génesis-8, showed 2.4 fold more upregulated DEGs than Williams-8 (1.028 versus 434 DEGs), while at 48 hpi the number of upregulated DEGs did not differ significantly between genotypes (1109 in Génesis-48 and 1205 in Williams-48). Comparative GO and KEGG pathway enrichment analysis resulted in similar terms and pathways in both infected genotypes, although Génesis-8 exhibited a greater number of genes in each category. Most enriched terms and pathways were related to plant defense, including defense response, response to oxidative stress and oxidant detoxification, phenylpropanoid and flavonoid biosynthesis, plant hormone signal transduction, plant-pathogen interaction, as well as protein phosphorylation and regulation of transcription. These findings suggest that an adequate recognition of the pathogen and activation of defense mechanisms may underlie the observed resistance in Génesis 5601 to *D. caulivora*.

The interplay between pathogen perception and defense activation has a profound effect on plant resistance. Our results revealed that 15 PRRs exhibited higher transcript levels in untreated Génesis 5601 compared to untreated Williams, including LRR-STKs, RLK and RLPs. This number increased to 59 upregulated PRRs in Génesis-8 versus 11 in William-8, including *RLKs*, *LRR-RLKs*, *LecRLKs, RLPs* and *CRKs*. Thus, basal PRRs expression levels as well as an early induction of PRRs upon *D. caulivora* inoculation might play important roles in MAMPs recognition and PTI activation. In accordance with these results, narrow-leafed lupin RLKs, LRR-RLKs, LecRLKs and RLP encoding genes were earlier induced in resistant compared to susceptible genotypes in response to *D. toxica* (Will., Highet, Gams, and Sivasith)^23^. Similarly, several PRR genes such as *CRKs* and *LRR-RLKs* were higher expressed in resistant compared to susceptible soybean plants in response to *Phakopsora pachyrhizi*^24^, soybean mosaic virus (SMV)^25^, and soybean cyst nematode (*Heterodera glycines* Ichinohe)^26^. Moreover, RLKs and RLPs are candidate soybean resistance genes against the fungus *Phialophora gregata* (sin. *Cadophora gregata*) (Allington & D.W. Chamb.) W. Gams ^27^.

Suppression of PTI by pathogen effectors activates ETI through the action of NLRs^10^. *D. caulivora* genome analysis evidenced the presence of 133 secreted effector candidates, and several of these effector genes were induced during soybean colonization, suggesting that they could interfere with soybean defense^16^. Here, we show that seven NLR genes exhibited higher expression levels in untreated Génesis 5601 versus Williams. Similarly, seven and only one *NLR* were upregulated in Génesis-8 and Williams-8, respectively. The induction of NLR genes could lead to earlier activation of downstream immune events in Génesis 5601. Soybean NLR genes co-localize with disease-resistance quantitative-trait loci (QTL)^28^, supporting their role in plant resistance against different pathogens. NLR were identified as candidate resistance genes for several soybean diseases caused by bacterial, fungal, oomycete and virus^29–36^. Some of these NLR genes have been functionally validated^31,37,38^. Furthermore, genomic regions identified by genome-wide association studies (GWAS) and related to resistance against several soybean diseases were enriched in LRR-RLK and NLR^39^.

MAPKs, MAPKKs, MAPKKKs and STKs activate downstream signaling after pathogen recognition^40^. *D. caulivora* inoculation increased expression of genes encoding for these type of kinases in both cultivars. Several *MAPKKKs* were only upregulated in Génesis-8 and STKs were differentially expressed in Génesis-8 and Williams-8. Interestingly, STKs are candidate genes for resistance to soybean mosaic virus and several *STKs* were higher expressed in resistant compared to susceptible plants^25^.

WRKY, AP2/ERF, MYB, bHLH, NAC, GRAS, and other TFs play important roles in defense responses to biotic and abiotic stress^41–43^. Our study found that 219 of these TFs were upregulated during *D. caulivora* colonization. Génesis-8 showed significantly more upregulated *ERFs*, *MYB* and *bHLH* compared to Williams-8, which could trigger coordinated induction of target genes involved in plant immunity. Similarly, higher expression levels of *bHLHs* were observed in *P. sojae* resistant compared to susceptible soybean cultivars^44^. Interestingly, GmMYBs and GmNAC regulate biosynthesis of flavonoids leading to the production of phytoalexins that increase resistance against pathogen^45–47^. Moreover, GmERF5- and GmERF113-overexpressing soybean plants showed enhanced resistance to *P. sojae* and positively regulated expression of PR-10 and PR-1^48,49^.

A high number of PR genes were induced during *D. caulivora* colonization in both cultivars, suggesting their involvement in soybean defense against this fungal pathogen. Likewise, PR-1, β-1,3-glucanase (PR-2), chitinases (PR-3 and PR-4), PR-10 and defensin were induced in a susceptible cultivar infected with *D. aspalathi*^13^. Expression profiles highlighted β-1,3-glucanase, peroxidases class III (PR-9) and PR-10 function in the early response of Génesis 5601. Similarly, peroxidases encoding genes were upregulated in a resistant genotype of narrow-leafed lupin infected with *D. toxica*^23^. As observed for other pathogens, these enzymes could protect plant cells against *D. caulivora* by degrading fungal cell wall polysaccharides (β-1,3-glucanases), and probably by inhibiting hyphal growth through RNase activity (PR-10)^50,51^. Peroxidases could increase soybean defenses by reinforcing plant cell walls, synthesis of phytoalexins, or participate in ROS metabolism as has been observed in other pathogen-infected plants^52^. In addition to peroxidases, we found increased expression of other oxidative stress-related genes encoding glutathione S-transferase, thioredoxin, ferredoxin oxidoreductases in Génesis-8, supporting the importance of cellular homeostasis to maintain a redox balance in soybean tissues to resist further infection. ROS accumulation can lead to a hypersensitive response (HR), a programmed localized cell death that occurs at the site of infection and is associated with restriction of the pathogen and disease resistance^53^. Interestingly, the induced expression of three genes encoding BAG cochaperone (Glyma.18G284900, Glyma.18G285100 and Glyma.07G061500) in Génesis-8, suggest a possible involvement of programmed cell death in *D. caulivora* resistance. This type of proteins trigger autophagy in the host to limit fungal colonization and confer resistance to fungal pathogens^54^.

Plants respond to pathogen infection by activating the phenylpropanoid pathway^14,55^. Here, we show that a high proportion of genes required for phenylpropanoid synthesis and lignin production were induced upon *D. caulivora* infection, suggesting that reinforcement of the cell wall through lignin and phenolic compounds, and synthesis of antimicrobial compounds such as flavonoids, isoflavonoids, coumarins and lignans are important defense mechanisms against this fungal pathogen. Interestingly, a significant number of upregulated DEGs were only present in Génesis-8 and not in Williams-8, suggesting that some of the metabolites produced by the phenylpropanoid pathway could be involved in resistance mechanisms against *D. caulivora*. Phytoalexin production in response to pathogens is regulated by the enzymes PAL, CHS, and chalcone isomerase (CHI), among others^14^. The number of upregulated DEGs encoding these enzymes were significantly higher in Génesis-8 compared to Williams-8. Interestingly, phenylpropanoids such as isoflavones daidzein, genistein and glyceollins are produced in soybean resistant plants after treatment with *D. aspalathi* elicitors^56^. Moreover, GmMYB29 regulates isoflavonoid biosynthesis in soybean through the activation of isoflavone synthase and CHS encoding genes^45^. Functional analysis demonstrated that GmMYB29A2 is crucial for the accumulation of glyceollin I and expression of *P. sojae* resistance^47^. Likewise, R2R3-MYB involved in lignin synthesis and genes responsive to chitin were significantly induced in *P. pachyrhizi* resistant genotypes^46^. Furthermore, activation of cell wall reinforcement by incorporation of phenolic compounds was previously observed in *D. caulivora* infected tissues^2^. Thus, these findings suggest that several metabolites of the phenylpropanoid could be produced during *D. caulivora* colonization, although further studies are needed to decipher the involvement of these metabolites in soybean resistance against this fungal pathogen.

Plant hormones conform a complex network that regulate plant resistance against pathogens^57^. Soybean plants activate hormonal pathways during *D. caulivora* infection in both genotypes. The significantly higher number of upregulated DEGs involved in IAA, ET, CK and JA pathways in Génesis-8 compared to Williams-8 suggests that these hormones could be involved in resistance responses against *D. caulivora*. Interestingly, induction of ACS and ACO genes involved in ET synthesis has been associated with resistance of soybean plants to vascular disease caused by *Fusarium virguliforme* (Link ex Grey)^58^. In addition, genes encoding enzymes involved in the production of JA, including LOXs and OPR, were earlier induced in *D. caulivora*-inoculated Génesis 5601 plants compared to Williams plants. Consistently, expression levels of LOXs increased in resistant genotypes of narrow-leafed lupin compared to susceptible genotypes in response to *D. toxica*^23^. Oxylipins produced by the LOX pathway play different roles during defense responses against biotic stress through antimicrobial activities, contribution to HR, and production of signaling molecules such as JA and related compounds that lead to induced expression of genes with multiple roles in defense^15,59^.

Small HSP are chaperones that play important roles in immunity by protecting cells from stress-induced protein aggregation and misfolding^60^. Remarkably, sHSPs encoding genes were only upregulated in Génesis 5601, and generally at 8 hpi, suggesting their important contribution in the early stages of plant resistance. HSPs are involved in stability and accumulation of PRRs and NLRs and further defense signaling^60,61^. In *Nicotiana tabacum*, a sHSP was shown to be involved in disease resistance against *Ralstonia solanacearum* (Smith)^62^. Furthermore, GmHsp22.4 was highly induced in a nematode resistant soybean genotype and its overexpression in Arabidopsis plants renders lower nematode multiplication^63^. Interestingly, some pathogen effectors interact with sHSPs to suppress chaperone activity and promote virulence^64^, highlighting the role of these plant chaperones in disease resistance.

## Conclusions

This comparative transcriptomic approach between contrasting soybean genotypes revealed that resistance of Génesis 5601 to *D. caulivora* infection could be related to induced expression of a higher number of genes encoding proteins involved in perception through PRR and NLR, as well as TFs, PRs, biosynthesis of phenylpropanoid derived metabolites, hormones, sHSPs and genes with different roles in defense. Future studies comprising functional characterization of soybean candidate genes and target genes of *D. caulivora* effectors will contribute to a valuable comprehension of soybean-*Diaporthe* interactions. These findings provide novel molecular insights into soybean defense mechanisms used to control this pathogen, and establish a foundation for improving resistance in breeding programs.

## Methods

### Plant materials and *D. caulivora* inoculation

For this study, we used the *D. caulivora* isolate D57, collected from canker lesions of soybean plants grown in Uruguay during 2015^2^. Two soybean genotypes were used for all plant assays: SSC-susceptible Williams PI548631 obtained from USDA ARS Soybean germplasm collection (seed source 13U-9280, order 253444, 2014), and the SSC-resistant Génesis-5601 from the Instituto Nacional de Investigación Agropecuaria (INIA) breeding program (Stewart S, personal communication). Three soybean seeds of each genotype were individually planted in a 10-cm-diameter pot filled with a mix of soil and vermiculite at a rate of 3:1. Soybean seedlings were grown in a growth room under a 16 h light/8 h dark photoperiod regime at 24°C. For all experiments, 3-week-old plants at V2 were used. *D. caulivora* D57 isolate was inoculated using the stem wounding method where an agar plug containing mycelium was applied to the wounded stem^2^. As a control an agar plug without mycelium was used. All experiments were performed with accordance to relevant regulations and guidelines.

Development of SSC symptoms were compared between both soybean genotypes. Ten plants were used per treatment and the experiment was repeated three times. Lesion length (mm) and disease severity (Scale 1–7), was determined at various time points (3, 5, 7, 11, and 14 dpi]. Disease severity index and area under disease progress curve (AUDPC) was calculated according to Mena et al.^2^. Differences between treatments were determined by non-parametric Kruskal–Wallis and Mann– Whitney tests using SPSS Statistics v. 21.0. P values of < 0.01 were considered as significant.

### Quantitative PCR

After soybean stem inoculation with *D. caulivora*, fungal DNA was quantified at 8, 24, 48, 72, and 96 hpi. Three plants per treatment were used as biological replicates and samples were frozen in liquid nitrogen. DNA was extracted from stem tissues (stem section of 1.5 cm including the wounded area) using the DNeasy kit (Qiagen, Germany). DNA concentration and quality were assessed using a NanoDrop 2000 spectrophotometer (Thermo Fisher Scientific, USA). Quantitative PCR (qPCR) was performed using primers designed for the elongation factor gene Ef1a of soybean (5′-GATTTCATGTAGCCGTAGCC-3′ and 5′-ATTTAAGACATCCCTCCTCAG-3′) and the β-tubulin gene of *D. caulivora* (5′-CCGTGGAAAGGTCTCTATGAAG-3′ and 5′-TCTGGACGTTGTTGGGAATC-3′). qPCR was performed using the QuantiNova Probe SYBR Green PCR Kit (Qiagen, Germany) in a 96-well thermocycler (New Applied Biosystems QuantStudio 3). Each reaction consisted in 20 μl containing 10 μl of SYBR Green PCR Master mix (2×), 0.7 mM primers mix, and DNA (~ 25 ng). The thermocycler was programmed to run for 2 min at 95°C, followed by 40 cycles of 15 s at 94°C and 20 s at 60°C. Water was used as negative control. As a standard, a serial dilution of genomic DNA from *D. caulivora* with known concentrations (60 ng, 6 ng, 600 pg, 60 pg, and 6 pg) were analyzed to determine the sensitivity and linear range of the assay. Pathogen standard curve was generated by plotting the CT values of a tenfold dilution series of *D. caulivora* DNA stock solution versus the logarithm of the concentration. The resulting regression equations were used to calculate fungal DNA in stem samples. Similarly, a standard curve was generated to estimate the amount of soybean DNA present in each sample. Pathogen β-tubulin estimated was expressed relative to soybean elongation factor. Each data point is the mean value of three biological replicates. Results were expressed as ng of *D. caulivora*/ng of soybean tissue. Student’s t-test was applied to all qPCR data, and values of p ≤ 0.01 were considered statistically significant.

### RNA extraction, cDNA library preparation and sequencing

Samples of both genotypes were taken from untreated plants, and from plants inoculated with plugs containing *D. caulivora* mycelium and their respective controls (plugs without mycelium) at 8 and 48 hpi. Total RNA was extracted and purified from 100 mg soybean stems, 10 mm above the inoculation point of each sample with TRIzol reagent (Invitrogen, Carlsbad, CA, USA) and Invitrogen PureLink RNA Extraction Mini kit (Invitrogen, USA), including an on-column digestion with RNase-Free DNase I, according to the manufacturer’s instructions. RNA quality was checked by running samples on 1.2% formaldehyde agarose gel. RNA concentration was measured using a NanoDrop 2000c (Thermo Scientific, Wilmington, USA). RNA quality control, library preparation, and sequencing were performed at Macrogen Inc. (Seoul, Korea). Three biological replicates were included per treatment. Libraries for each biological replicate were prepared for paired-end sequencing by TruSeq Stranded Total RNA LT Sample Prep Kit (Plant) with 1 μg input RNA, following the TruSeq Stranded Total RNA Sample Prep Guide, Part # 15031048 Rev. E. Sequencing was performed on Illumina platform (Illumina, CA, USA) to generate paired-end 101 bp reads, obtaining 41.6 to 65.2 M reads per sample with Q20 > 98% and Q30 > 95%.

### Pre-processing of raw data, mapping of reads and annotation

RNA-seq processing steps were done through Galaxy platform (https://usegalaxy.org/)^65^ and according to Reboledo et al.^66^. Raw reads quality was subjected to a quality control check using FastQC software ver. 0.11.2 (http://www.bioinformatics.babraham.ac.uk/projects/fastqc/). Sequences were trimmed, and the adapters removed using Trimmomatic Version 0.38.0 software^67^. Additionally, to the default options, the following parameters were adjusted: adapter sequence TruSeq3 (paired-ended (PE), for MiSeq and HiSeq), always kept both PE reads, and SLIDINGWINDOW: 4:15 HEADCROP: 13 MINLEN:50. Trimmed reads were mapped to reference genome of *Glycine max* Gmax_275_Wm82.a2.v1.fa^68^ as the reference genome file and Gmax_275_Wm82.a2.v1.gene.gff3 as a reference file for annotation gene models from Phytozome (https://phytozome.jgi.doe.gov/pz/portal.html) using Hisat2 software^69^. The BAM files were obtained with Samtools View software ver.1.9 and then sorted by name with Samtools Sort software ver. 2.0.3^70^, for further analysis.

### Differential gene expression and functional analysis

Cluster analysis of replicates from each time point and control samples were performed by Principal Component Analysis (PCA) using log2 fold changes for all datasets. Reads were counted using FeatureCounts software ver. 1.6.4^71^. Additionally, to default options, the following parameters were set: Stranded (Reverse), Count fragments instead of reads -p, Allow read to map to multiple features True, Count multi-mapping reads/fragments -M and Reference sequence file *Glycine max* Wm82.a2.v1. Differential expression analyses were performed using EdgeR software ver. 3.24.1^72^, with p-value adjusted method of Benjamini and Hochberg adjusted threshold 0.05^73^, and Minimum log2 Fold Change 2. Counts were normalized to counts per million (cpm) with the TMM method and low expressed genes filtered for count values ≥ 3 in all samples. In this study, a false discovery rate (FDR) ≤ 0.05 was used to determine significant differentially expressed genes (DEGs) between *D. caulivora* inoculated plants and mock; and expression values were represented by log2 ratio. Heat maps were generated using the Heatmapper server (https://www.heatmapper.ca/expression). Hierarchical clustering analysis of expressed genes were performed on log2 Fold-Change expression values using the “hclust” tool from R package “stats” ver. 3.6.0. To visualize the obtained data, heatmap plots were performed using the “heatmap.2” tool from R package “gplots” ver. 3.1.0.

Gene ontology (GO) and functional annotations were assigned with the Blast2GO, through Omicbox software (https://www.biobam.com/omicsbox)^74^. Gene models were compared with several databases (NCBI nonredundant protein database, GO, and InterpoScan) with BlastP finding single hit at an e-value threshold of e-value ≤ 1.0E−3 using taxIds for Viridiplantae. InterproScan analysis was used to identify domains in the genome^75^ DEG functional enrichment analysis was performed using OmicBox software. GO terms with a FDR ≤ 0.05 were considered for the analysis. DEGs of each dataset were divided into upregulated and downregulated subsets. Kyoto Encyclopedia of Genes and Genomes (KEGG) enrichment analysis of all DEGs were obtained through Omicbox software for *D. caulivora* inoculated vs. control samples in both soybean genotypes. All heat maps were generated using the Heatmapper server (https://www.heatmapper.ca/expression).

### Quantitative real-time PCR

The expression level of twenty-four selected candidate genes was analyzed via quantitative reverse transcription PCR (RT-qPCR). cDNA was generated from 1 μg of RNA using RevertAid Reverse transcriptase (Thermo Scientific) and oligo (dT) according to the manufacturer’s protocol. RT-qPCR was performed in an Applied Biosystems QuantStudio 3 thermocycler using the QuantiNova Probe SYBR Green PCR Kit (Qiagen, Germany); mix proportions and cycling parameters were used as described in manufacturer’s instructions. Relative expression of each gene was normalized to the quantity of constitutively expressed elongation factor 1-alpha gene, using the 2−ΔΔCt method^76^. Gene expression of soybean-inoculated tissues was expressed relative to the corresponding control samples at the indicated time points, with its expression level set to one. Each data point is the mean value of three biological replicates. Student’s t-test was performed to determine the significance for quantitative gene expression analysis using GraphPad Prism software ver. 8.0.1. P-values < 0.01 were considered statistically significant. Primer pairs used for qPCR analyses are provided in Supplementary Table S12. In all cases, amplification efficiencies were between 95 and 110%.

### Supplementary Information

Supplementary Figure S1.

Supplementary Figure S2.

Supplementary Figure S3.

Supplementary Figure S4.

Supplementary Table S1.

Supplementary Table S2.

Supplementary Table S3.

Supplementary Table S4.

Supplementary Table S5.

Supplementary Table S6.

Supplementary Table S7.

Supplementary Table S8.

Supplementary Table S9.

Supplementary Table S10.

Supplementary Table S11.

Supplementary Table S12.

Supplementary Legends.

## Data Availability

RNA sequencing data were deposited at the National Center for Biotechnology Information (NCBI) in the Sequence Read Archive (SRA) under the PRJNA878492 Bioproject accession.
