# Supplementary figures and images for: Comparative analysis of soybean transcriptional profiles reveals defense mechanisms involved in resistance against Diaporthe caulivora
Source: Sci Rep. 2023 Aug 11;13:13061. doi: 10.1038/s41598-023-39695-1 (PMC10421924; doi:10.1038/s41598-023-39695-1)

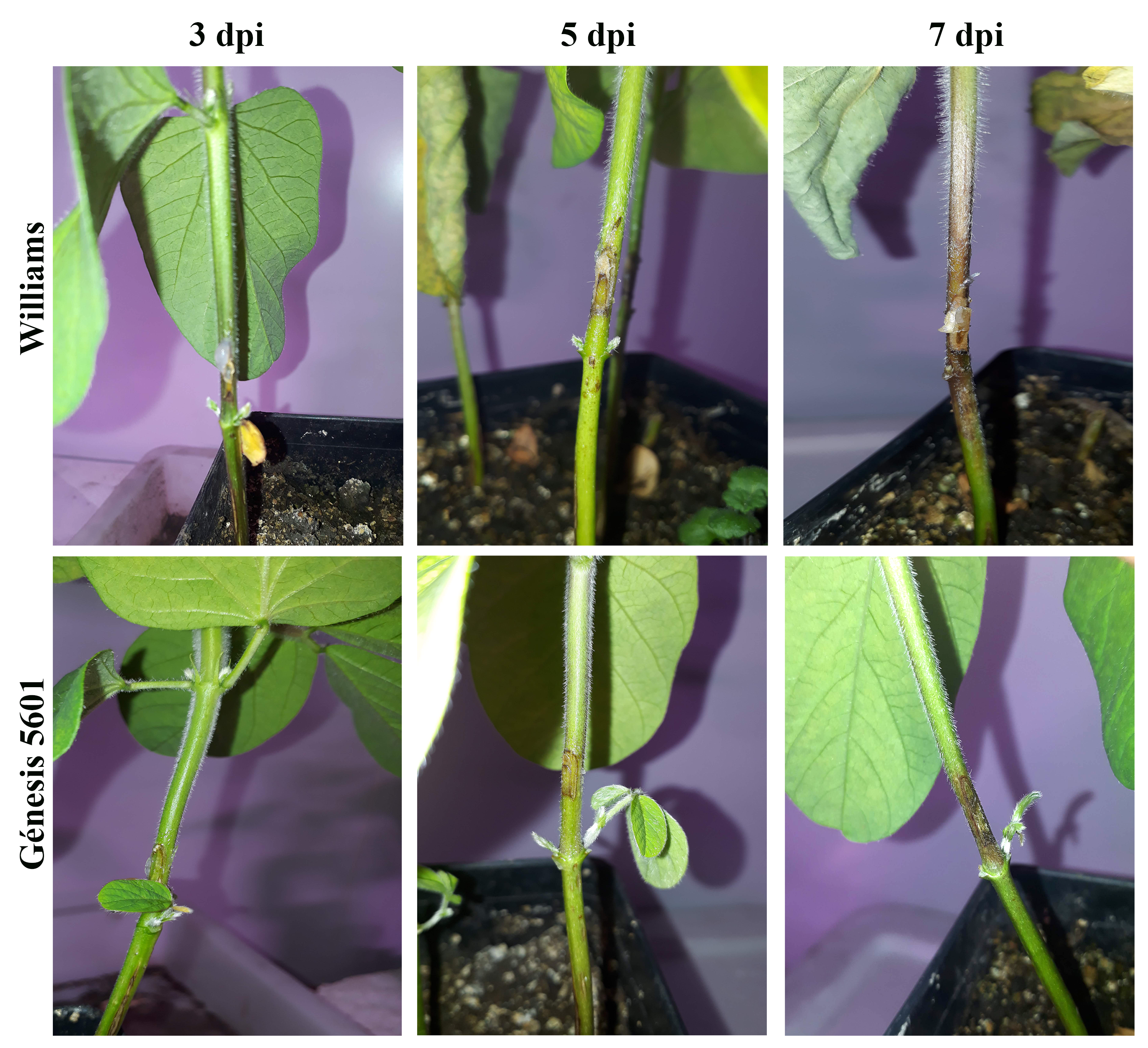

Supplement: Supplementary file 1 — Supplementary Figure S1. [file 41598_2023_39695_MOESM1_ESM.tif]

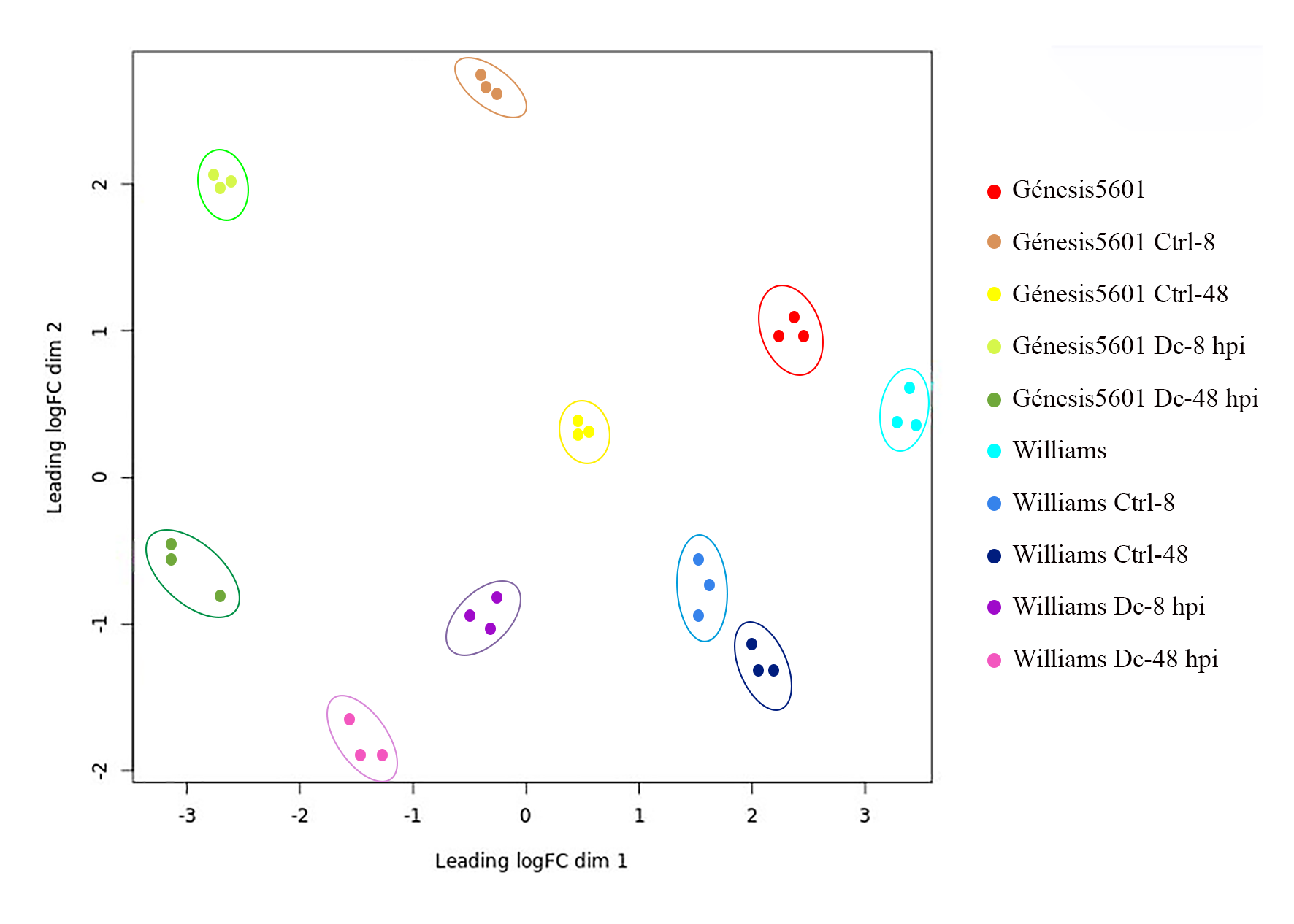

Supplement: Supplementary file 2 — Supplementary Figure S2. [file 41598_2023_39695_MOESM2_ESM.tif]

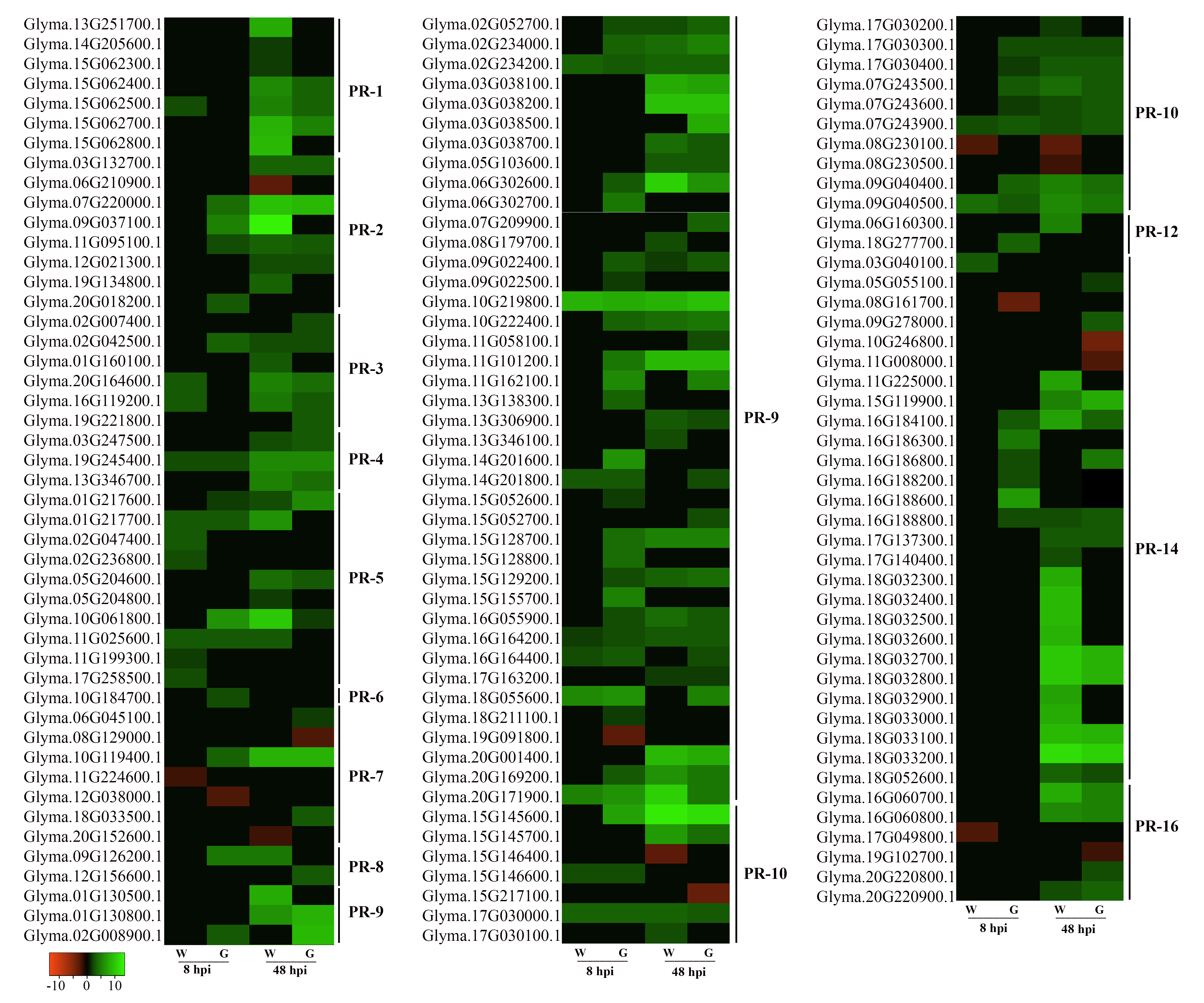

Supplement: Supplementary file 3 — Supplementary Figure S3. [file 41598_2023_39695_MOESM3_ESM.tif]

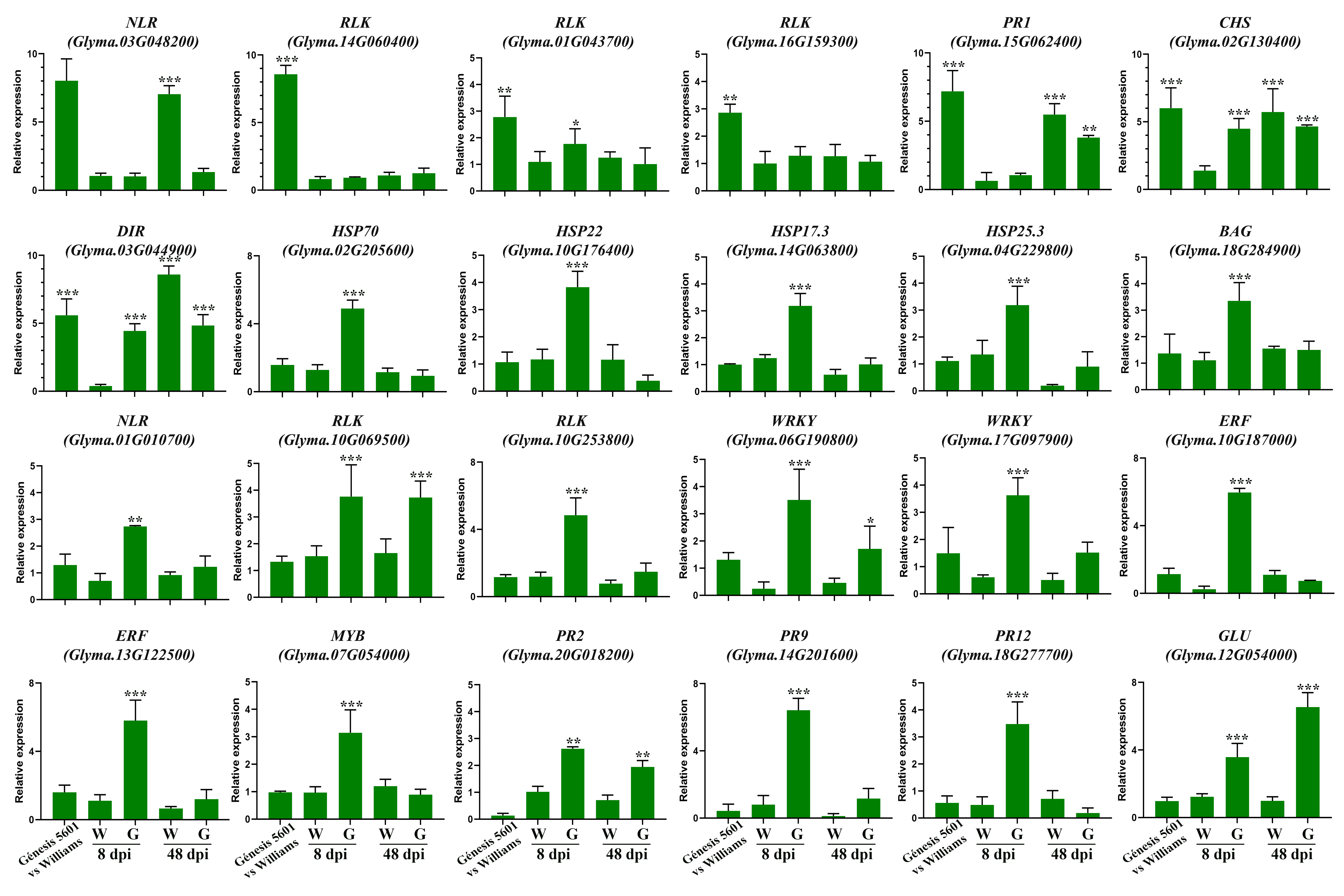

Supplement: Supplementary file 4 — Supplementary Figure S4. [file 41598_2023_39695_MOESM4_ESM.tif]
