# Supplementary material for: Comparative analysis of soybean transcriptional profiles reveals defense mechanisms involved in resistance against Diaporthe caulivora
Source: Sci Rep. 2023 Aug 11;13:13061. doi: 10.1038/s41598-023-39695-1 (PMC10421924; doi:10.1038/s41598-023-39695-1)
Supplement: Supplementary file 17 — Supplementary Legends. [file 41598_2023_39695_MOESM17_ESM.docx]

**Supplementary Legends**

**Supplementary Figure S1.** Soybean stem canker disease progress after *D. caulivora* inoculation at 3, 5 and 7 days post-inoculation (dpi).

**Supplementary Figure S2.** Two-dimensional scatterplot of the principal component analyses (PCA) for soybeans where distances approximate the typical log2 fold changes between the samples. Colored dots denote each biological replicate.

**Supplementary Figure S3. Heatmap of differentially expressed genes (DEGs) encoding for pathogenesis-related proteins (PRs).** Individual genes are listed and colors represent the log2 fold change value based on the comparison of the transcript levels between *D. caulivora*-inoculated and control treatment for both genotypes (Williams and Génesis 5601). Green represents upregulated DEGs and red downregulated DEGs. Abbreviations: pathogenesis-related protein 1 (PR-1), glucan endo-1,3-beta-glucosidase (PR-2), chitinases I and II (PR-3, PR-4), thaumatin-like (PR-5), proteinase inhibitor (PR-6), endoproteinase (PR-7), chitinase III (PR-8), peroxidases (PR-9), ribonuclease-like (PR-10), defensin (PR-12), lipid transfer proteins (PR-14), and oxalate-oxidase-like or germin-like (PR-16). See Supplementary table S8 for complete information.

**Supplementary Figure S4**. Validation of soybean differentially expressed genes in untreated plants and during infection with *D. caulivora* at 8 and 48 hpi by quantity reverse transcription RT-qPCR. The expression levels of soybean genes at the indicated time points are relative to the level of expression of soybean genes in control plants, using elongation factor 1-alpha as reference gene. Results are reported as means ± standard deviation (SD) of three samples for each treatment. Asterisks indicate a statistically significant difference between soybeans genes in plants inoculated with *D. caulivora* and control plants. Asterisks indicate a statistically significant difference between gene expression in plants (Student’s t-test, * p < 0.5, ** p <0.01; *** p < 0.005), Abbreviation: nd: no data. See Supplementary table S2 for complete information.

**Supplementary Table S1:** Summary of mapped reads of Williams and Génesis 5601 RNA-Seq libraries. 1-3 indicate the three biological replicates in control stems and during *D. caulivora* stem infection at the indicated time points.

**Supplementary Table S2.** List of soybean differentially expressed genes (DEGs) and Gene Ontology (GO) terms. Comparisons were performed between untreated Génesis 5601 versus untreated Williams (G-W), and *D. caulivora* inoculated versus control plants of both genotypes at the indicated time points (Gi8-G8; Gi48-G48; Wi8-W8; Wi48-W48).

**Supplementary Table S3:** Kyoto Encyclopedia of Genes and Genomes (KEGG) pathway analysis of upregulated genes in soybean plants inoculated with *D. caulivora*.

**Supplementary Table S4:** Hierarchical clustering of all DEGs in soybean plants inoculated with *D. caulivora*.

**Supplementary Table S5:** List of Gene Ontology (GO) enrichment terms obtained for soybean differentially expressed genes present in the different clusters.

**Supplementary Table S6:** List of DEGs encoding for proteins with role in perception and signaling during *D. caulivora* infection in both genotypes at 8 and 48 hpi.

**Supplementary Table S7:** List of DEGs encoding for proteins involved in transcription during *D. caulivora* infection in both genotypes at 8 and 48 hpi.

**Supplementary Table S8:** List of DEGs encoding for pathogenesis-related proteins during *D. caulivora* infection in both genotypes at 8 and 48 hpi.

**Supplementary Table S9:** List of DEGs encoding for proteins involved in phenylpropanoids and flavonoids pathways during *D. caulivora* infection in both genotypes at 8 and 48 hpi.

**Supplementary Table S10:** List of DEGs involved in hormone signaling during *D. caulivora* infection in both genotypes at 8 and 48 hpi.

**Supplementary Table S11:** S5: Validation of RNA-Seq data by qPCR assay and correlation of log2 FC values for 24 genes obtained by RNA-Seq and qPCR. Gene name and identification, log2 fold change from RNA-seq and RT-qPCR analysis and description.

**Supplementary Table S12:** List of qPCR primers used in this study.
